# Environmental chemicals, breast cancer progression and drug resistance

**DOI:** 10.1186/s12940-020-00670-2

**Published:** 2020-11-17

**Authors:** Meriem Koual, Céline Tomkiewicz, German Cano-Sancho, Jean-Philippe Antignac, Anne-Sophie Bats, Xavier Coumoul

**Affiliations:** 1 INSERM UMR-S1124, 3TS, Toxicologie Pharmacologie et Signalisation Cellulaire, Université de Paris, Paris, France; 2 Assistance Publique-Hôpitaux de Paris, Hôpital Européen Georges-Pompidou, Service de Chirurgie Cancérologique Gynécologique et du Sein, Paris, France; 3 Faculté de Médecine, Université de Paris, Paris, France; 4 LABERCA, Oniris, INRA, Université Bretagne-Loire, 44307 Nantes, France; 5 INSERM UMR-S1147, Equipe labellisée Ligue Nationale Contre le Cancer, Université de Paris, Paris, France

**Keywords:** Breast cancer, Environmental exposure, Organochlorine pesticides, Endocrine disrupting chemicals, Polychlorinated biphenyls, Perfluoroalkyl acid, Environmental pollutants; aryl hydrocarbon receptor; cancer stem cells; drug resistance

## Abstract

Breast cancer (BC) is one of the most common causes of cancer in the world and the second leading cause of cancer deaths among women. Mortality is associated mainly with the development of metastases. Identification of the mechanisms involved in metastasis formation is, therefore, a major public health issue. Among the proposed risk factors, chemical environment and pollution are increasingly suggested to have an effect on the signaling pathways involved in metastatic tumor cells emergence and progression. The purpose of this article is to summarize current knowledge about the role of environmental chemicals in breast cancer progression, metastasis formation and resistance to chemotherapy. Through a scoping review, we highlight the effects of a wide variety of environmental toxicants, including persistent organic pollutants and endocrine disruptors, on invasion mechanisms and metastatic processes in BC. We identified the epithelial-to-mesenchymal transition and cancer-stemness (the stem cell-like phenotype in tumors), two mechanisms suspected of playing key roles in the development of metastases and linked to chemoresistance, as potential targets of contaminants. We discuss then the recently described pro-migratory and pro-invasive Ah receptor signaling pathway and conclude that his role in BC progression is still controversial. In conclusion, although several pertinent pathways for the effects of xenobiotics have been identified, the mechanisms of actions for multiple other molecules remain to be established. The integral role of xenobiotics in the exposome in BC needs to be further explored through additional relevant epidemiological studies that can be extended to molecular mechanisms.

**Supplementary Information:**

The online version contains supplementary material available at 10.1186/s12940-020-00670-2.

## Background

The increasingly high incidence of breast cancer (BC) in women is a major public health concern. Even if the prognosis is excellent when the cancer is located in the breast (5-year survival rate, 99%), survival rates decrease rapidly in the case of disseminated disease (26% survival rate if distant metastasis is present [1, 2]). Moreover, drug-resistance to chemotherapy, especially in some subtypes of BC, presents a great challenge to clinicians striving to improve survival of BC patients. Cancer progression in the last phase of tumor development (which can occur in cases of drug-resistance) is defined by an increased speed in growth and invasiveness of the tumor cells. This leads to the acquisition of metastatic potential in cancer cells. Metastasis involves the spread of cancer cells from the primary tumor to surrounding tissues and to distant organs. It is the primary cause of cancer morbidity and mortality and is responsible for about 90% of cancer deaths [3]. Although many types of cancers are susceptible initially to chemotherapy, over time they can develop resistance through diverse mechanisms, such as DNA mutations and metabolic changes that promote drug inhibition and degradation [4].

Exposures to environmental chemicals are ubiquitous. During our lives, we are exposed to many known toxicants as well as to a number of potentially hazardous chemicals which have less well-characterized risks. Plastic food and beverage containers, cosmetics, sunscreen, cleaning products and garden products all contain chemicals. Chemical pesticides are residues on many commercially grown fruits and vegetables and grain crops. Moreover, several chemicals are defined as persistent organic pollutants (POPs) on the basis of their resistance to degradation, their environmental persistence and their bioaccumulation in the food chain. They have been banned for decades in most countries because of human health concerns. However, they still accumulate in soils, sediments, air and biota because of their long half-lives. Human beings are still exposed to these chemicals through several routes [5].

Increasing epidemiological evidence, as well as a better understanding of the mechanisms which link toxicants to the development of cancer suggest that exposures to some environmental chemicals found in common products may lead to an increased risk of developing cancer. More recently, the role of exposure to low-doses of environmental pollutants in cancer initiation as well as in cancer progression has been raised by several studies which suggest that these chemicals may promote cancer invasion and metastasis [6]. Thus, the purpose of this review is to summarize the main findings related to the role of environmental contaminants on the promotion of invasion and metastasis in BC and their link(s) with chemoresistance.

## Method

We present here a scoping review of the evidence for chemicals associated with invasion and metastasis in BC as well as their association with resistance to chemotherapy. We searched PubMed for peer-reviewed articles published before December 2018 that reported on studies of BC, environmental pollutants and cancer spreading. We included in vitro and in vivo animal studies as well as epidemiological studies. Searches included the MESH term “breast neoplasm” (and synonyms as “breast cancer” and “breast carcinoma”) in combination with the terms for specific chemicals, chemicals groups and product classes that were used in two previous published reviews [7, 8] along with the following terms linked to cancer progression and aggressivity: “cancer proliferation”, “cancer invasion”, “cancer invasiveness”, “cancer spreading”, “metastasis formation”, or “resistance to chemotherapy”, “chemoresistance”, “resistance to cancer treatment”, “drug resistance”. The search terms for chemicals included the chemicals investigated in the recent review by Rodgers published in 2018 [8]. Included are chemicals identified as mammary carcinogens by the Unites States National Toxicology Program (NTP, available online https://ntp.niehs.nih.gov) or classified as potential mammary carcinogens with substantial population exposure and chemicals identified as mammary gland developmental disruptors. The terms for consumer products, alcohol, cigarette smoke and toxic metals also have been included in the search. The complete list of the chemicals investigated is available in Table S1 [8].

## Results of review

**Fig. 1:**
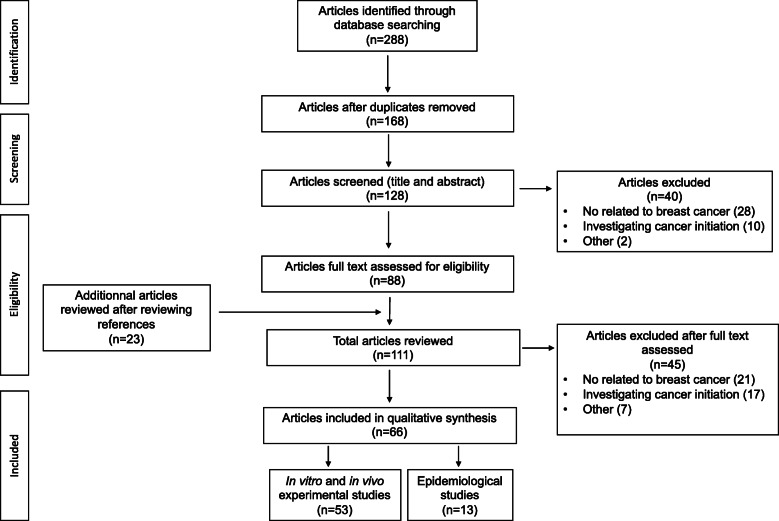
Flow diagram of the study selection

**Fig. 2:**
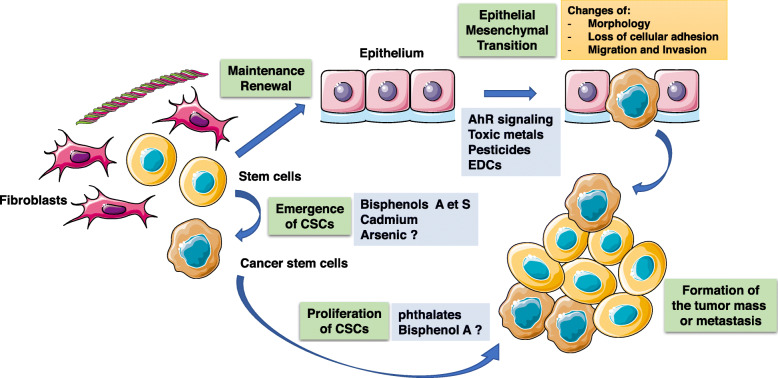
Epithelial-to-mesenchymal transition and appearance of cancer stem cells are two mechanisms which are suspected to lead to the occurrence of metastasis. EMT is associated to the phenotypical acquisition of cellular properties which leads to the migration and invasion of primary tumor cells while cancer stem cells conserve cellular properties of stem cells (important for maintenance and renewal of the breast cellular epithelium) which maintain cancer proliferation, metastatic dissemination and resistance to anticancer treatment. The figure also synthesized the processes which are targeted by environmental pollutants (orange squares). This figure was drawn using the website « Smart Servier Medical Art » (https://smart.servier.com/)

Two hundred eighty-eight articles were identified through database searching. After removing duplicates, articles not focused on BC and articles dealing with cancer initiation, 88 relevant articles which investigated the role of chemicals in BC progression were retained for full text assessment. Twenty-three additional pertinent references were identified in the articles reviewed. Finally, 66 articles were retained for qualitative synthesis: 53 articles dealing with in vitro and in vivo experiments and 13 epidemiological studies. The information flow diagram for the selection of articles including in the scoping review the is presented in Fig. 1. The findings for each chemical of interest or chemical class are described successively. The main findings along with the suspected targeted signal pathway or mechanisms are summarized in Table 1 and Fig. 2 and epidemiological studies are described in Table 2.

**Table 1:** Breast cancer findings for environmental chemical disruptors in experimental studies that investigated cancer progression and targeted signal pathways

Potential environmental chemical disruptors	Studies (ref)	Type of study (in vitro/in vivo)	Effects that chemicals may have	Targeted signal pathways
**Persistent EDCs**				
Dioxin	[9]	In vivo	↓ tumor growth in immature 120-150 g female Sprague-Dawley rat *(no more precision)*	Unestablished
	[10]	In vitro	↓ invasion, motility and colony formation (MDA-MB-231, MCF7, ZR75, SKBR3) ↑ differenciation in a putative mammary cancer stem cell line	AhR dependant pathway regardless to ER status
	[11]	In vivo*/* In vitro	↓ proliferation (MCF7) ↓ tumor growth in male B6D2F mice (	Antagonistic effect on ER signaling
	[12]	In vitro	↓ proliferation (MCF7, TD47, ZR75)	Antagonistic effect on ER signaling
	[13]	In vitro	↓ migration (MCF7)	Downregulation of CXCR4 and CXCL12
	[14]	In vivo*/* In vitro	↓ metastasis formation in BALB/C mice *(from NCI Charles Rivers, Frederick, MD, USA)* No effect on tumor growth nor cell proliferation	Unestablished
	[15]	In vitro	↑ cell migration	NFATc1/ATX-signaling pathway
	[16]	In vitro	EMT, ↑ migration	AhR pathway
	[17]	In vitro	↑ invasion Resistance to apoptosis Mitochondrial dysfonction (↑ cytosolic [Ca(2+)](c) and RyR1-specific Ca(2+) release, ↑ calcineurin (CnA) levels and activation of its factors.	Mitochondrial transmembrane potential disruption in a time-dependent way Mitochondrial transcription and translation inhibition Activation of CnA-sensitive NF-kappaB/Rel (IkappaBbeta-dependent) factors.
	[18]	In vivo*/* In vitro	↓ colony formation (BP1, Hs578T, SUM149) ↑ migration ↓ metastasis (2-days zebrafish larvae AB x Fli-GFP)	AhR signaling pathway
Polychlorinated biphenyls	[19]	In vivo*/* In vitro	↑ migration (MCF7, MDA-MB-231 ↑ growth tumor and metastasis in NOD SCID immune- deficient mice *(no more precision)*	ROCK signaling pathway
	[20]	In vitro	↑ transendothelial migration (MDA-MB-231)	Overexpression of VEGF through PI3K pathway signaling
	[21]		↑ transendothelial migration, ↑MMP3 (human endothelial cells)	Activation EGFR and JAK3 in a coordinated and cross-regulated way AK3 and EGFR stimulate in concert PCB-induced activation of JNK and ERK1/2 followed by increased DNA binding of AP-1 and PEA3 and transcriptional up-regulation of MMP-3 expression.
**DDT/DDE and organochlorine pesticides**				
DDT/DDE	[22]	In vivo	↑ ER + tumor growth in 150 g Wistar Furth ovariectomized rat *(from Harlan Sprague Dawley, Madison, WI)* ↑ proliferation MT2 and MTW9/PL estrogen responsive mammary adenocarcinoma	ER signaling pathway Estrogen-androgen balance disruption
	[23]	In vitro	↑ proliferation in CAMA-1 human ER+ breast cancer cells	Opposing androgen signalling pathway that inhibits growth in hormone-responsive
Hexachlorobenzene	[24]	In vitro	↑ proliferation (MCF7)	IGF-I) signaling pathway
	[25]	In vitro	↑ migration (MDA-MB-231)	c-Src/HER1/STAT5b and HER1/ERK1/2 signaling pathways
	[26]	In vivo*/* In vitro	↑ invasion and MMP2/9 (MDA-MB-231) ↑ metastasis in mice (regardless ER status) in nude female Swiss BALB/C mice *(La Plata Laboratory Animal Facility, Buenos Aires, Argentina)*	AhR, c-Src, HER1, STAT5b, and ERK1/2 signaling pathways
	[27]	In vitro	↑ migration and invasion	Modulation of the crosstalk between AhR and TGFβ signaling
**Consumer product chemicals**				
Bisphenol A	[28]	In vitro	↑ expression MMP2/ MMP9 in TNBC –triple negative breast cancer (MDA-MB-231 and BT-549)	Activation ERRγ through ERK1/2 and Akt pathway
	[29]	In vitro	↑ proliferation and invasion in TNBC –triple negative breast cancer (MDA-MB-231 and BT-549)	Unestablished
	[30, 31]	In vitro	↑ proliferation (MCF7)	Upregulation of cell cycle genes Downregulation of antiproliferative genes
	[32]	In vitro	Alteration of the expression of cell cycle related genes	Activation of Estrogen Receptor dependent signaling pathway
	[33]	In vitro	↑ proliferation Induction of a profile of tumor aggressiveness in high-risk cells from breast cancer patients	Unestablished
	[34, 35]	In vitro	↑ migration and invasion (MDA-MB-231)	Activation GPER dependant pathway Activation of FAK, Src, ERK2-dependant pathway
Phtalates	[36]	In vitro	Phenotypical and gene expression changes associated with EMT (R2d cells, stem cell derived human breast epithelial cell line)	Activation of EGFR-PKA signaling cascade that increase AP2a transcriptor factor which upregulate histone deacetylase 3
	[37]	In vitro*/*In vivo	↑ proliferation, migration and colony formation (MDA-MB-231) ↑ tumor formation in Female nude mice BALB/cAnN.Cg-Foxn1nu/CrlNarl, 4–6 wk. old *(from the National Laboratory Animal Center Taipei, Taiwan)*	AhR/HDAC6/c-Myc signaling pathway
	[38]	In vitro	↑ proliferation (MCF7)	Activation of PPARα and γ
	[39]	In vitro	↑ proliferation (MCF7)	P13K/AKT signaling pathway
	[40]	In vitro	↓ tamoxifen-induced apoptosis in ER+ cells (MCF7) but not ER- cells (MDA-MB-231	Increased Bcl-2 to Bax ratio through an Estrogen Receptor dependent signaling pathway
Benzophénone-1/Nonylphenol	[41]	In vitro	↑ proliferation and migration (MCF7)	Upregulation of cyclin D1 and cathepsin D and downregulation in p21 regulated by an ERα-dependent pathway
Per and polyfluoroalkyl acids				
PFOA	[42]	In vitro	↑ proliferation, migration and invasion (MCF10)	Upregulation of cyclin D1 and CDK4/6 and downregulation and in p27 through PPARα-dependant pathway
PFOS	[43]	In vitro	↑ proliferation, migration and invasion (MCF10)	Upregulation of CDK4 and down regulation and downregulation in p27, p21 and p53
**Food preparation**				
Benzo(A)pyrene	[35]	In vitro	↑ migration (MDA-MB-231, MCF7) ↑ αvβ3 integrin-cell surface levels and an increase of metalloproteinase (MMP)-2 and MMP-9	Lipoxygenase- and Src-dependent pathway Activation of FAK, Src and extracellular signal-regulated kinase 2
	[44]	In vitro	↑ invasion (MDA-MB-231)	Upregulation of COX II and PGE2 through an AhR signaling pathway
	[45]	In vivo*/*In vitro	↑ migration and invasion, ↑MMP9 (MCF7) ↑ growth tumor and liver and lung metastasis in an accumulative mouse model mimicking the cumulative effects of chronic BaP exposure in female BALB/C mice (*from the Shanghai Laboratory Animal, China)*	Upregulation of ROS-induced ERK signaling pathway
2-amino-1-methyl-6-phenylimidazo[4,5-b]pyridine	[46]	In vitro	↑ migration and invasion, ↑ MMP9 (MCF7, TD47)	Upregulation of Cathepsin D and cyclooxygenase 2 through ER signaling
	[47]	In vivo*/*In vitro	Carcinogenesis effects, ↑ proliferation, migration, invasion, ↑ colony formation and stem-like cell populations (MCF10) ↑tumorigenicity, ↑ lung metastasis in athymic NCS-nu/nu mice (*(no more precision)*	Ras-ERK-Nox-ROS signaling pathway
**Other polycyclic aromatic carbons**				
Cigarette smoke	[48]	In vivo	↑ total pulmonary metastatic burden in smoke-exposed animals (female sexually mature BALB/cAnN mice) *(from Charles River Laboratories, Wilmington, MA, USA)*	Unestablished
	[49]	In vitro*/* In vivo	Phenotypical and gene expression changes associated with EMT (MCF7) Emergence of stem-like cells population, colony formation ↓ tumor size ↑ lung metastasis and liver cancer cells in female immuno deficient NSG mice *(from The Jackson Laboratory, Bar Harbor, ME, USA)*	Unestablished Activation of nAChRs, Src and calcium-dependent signaling pathway
	[50]	In vitro	↑ proliferation and invasion ↑ migration in a dose-dependent manner Phenotypical and gene expression changes associated with EMT (MCF7 and MDA-MB-468)	Unestablished
	[51]	In vitro	↑ proliferation ↑ of stem-like cells population Resistance to Doxorubicin	
7,12-dimethylbenz(a)anthracene	[52]	In vitro	↑ proliferation and invasion (mice) ↑ colony formation EMT (↓ E cadherin)	NFκB pathway
**Alcohol**	[53] (review)	In vitro*/* in vivo	↑Angiogenesis, migration, invasion, EMT, MMP ↑ Cancer stem cells ↑ Metastasis formation in MMTV neu transgenic mice *(no more precision)*	Cross-talk between oxidative stress and EGFR/ErbB2 signaling
**Toxic metals**	[54]	In vitro	Cadmium ↑ migration and invasion (MCF7, MDA-MB-231)	TGIF/MMP2 signaling axis
	[55]	In vivo*/* In vitro	Tungsten ↓ primary tumor growth but ↑metastasis in female BALB/C mice No change observed in invasiveness of cells in vitro (66Cl4 model of breast cancer metastasis to bone) *(from Charles Rivers Laboratories, Montréal, Canada)*	Targeting microenvironment: activation CAFs at the metastatic site (↑ MMP9) and ↑ of myeloid-derived suppressor cells

**Table 2:** Summary of epidemiological studies investigating exposure to environmental chemical disruptors and breast cancer aggressivity/progression or survival

Year/Country	Studies (ref)	Study design	Study population (percentage premenopausal)	Type of chemicals investigated	Measurment of exposure	Time at measurement	Outcome(s)	Results - hazard ratios	Conclusion/Comments
2000 Danemark	[69]	Cohort	195 women with breast cancer	Organochlorines	Serum Dieldrin HCB DDE, DDT and their metabolites 27 PCB (28, 42, 66, 74, 99, 101, 105, 110, 118, 138,146, 153, 156, 170, 172, 177, 178, 180, 183, 187, 189, 193,194, 195, 201, 203, and 206)	At diagnosis; from 1976 to 1978	Association DDT and DDE levels and breast cancer survival	Dieldrin exposure was associated with a decrease overall survival OR = 4.6 (95% CI = 1.8–11.5)	Exposure to estrogenic organochlorines such as dieldrin may affect breast cancer survival
2000 Canada	[58]	Case control	315 women newly diagnosed with breast cancer 307 controls	Organochlorines	Serum 14 PCB congeners (28,52,99,101,105,118,128,138,153,156,170,180,183,187) 11 chrorinated pesticides or their metabolites (aldrin, α-chlordane, γ-chlordane, p,p′-DDT, p,p′-DDE, hexachlorobenzene, β-HCH, mirex, cis-nonachlor, trans-nonachlor, oxychlordane)	At diagnosis; from 1994 to 1997	Association between organochlorine concentrations and tumor size and axillary lymph node involvement	↑ risk of lymph node involvement with exposure to p,p′-DDE OR = 2.54 (95% CI = 1.20–5.35) (comparison between highest and lowest tertiles) Dose related increased risk for DDE and large tumors withlymph node involvement Associations between tumor size and lymph-node involvement for betaHCH, oxychlordane, and trans-nonachlor ↑ risk of lymph node involvement with PCB 153 (chosen as a surrogate for all the highly prevalent PCBs) OR = 2.12 (95% CI = 1.5–4.30) No interaction between organochlorine exposure and the hormonal status of the tumor with regard to either axillary-lymph-node involvement or tumor size (204 patients)	p,p’-DDE and PCB 153 exposure could be associated with a more aggressive breast cancer phenotype regardless of ER status
2001 USA	[80]	Case control	88 women with unilateral invasive breast cancer and pulmonary metastatic disease matched with 176 controls patients without metastatic disease	Tabacco smoke	Smoking “dose” not available No chemicals measured	At diagnosis; no information about period	Association between cigarette smoking and the development of pulmonary metastatic disease	−24.1% in case patients active smokers vs 15.3% in control Unadjusted OR = 1.76 (no IC), *p* = 0,06 -Multivariate analysis including hormonal therapy and presence of other metastatic sites: OR = 1,96 (95%IC = 0.96–4.02), *p* = 0.06	Cigarette smoking could increase pulmonary metastatic disease risk
2001 Danemark	[59]	Case control	240 women with breast cancer and 477 controls	Organochlorines	Dieldrin HCB 27 PCB (28, 42, 66, 74, 99, 101, 105, 110, 118, 138,146, 153, 156, 170, 172, 177, 178, 180, 183, 187, 189, 193,194, 195, 201, 203, and 206)	At diagnosis; from 1976 to 1978	Association between organochlorine concentrations and breast cancer risk and survival according to estrogen receptor status	Sum of PCB was significantly associated with risk of death among women with ER+ tumors OR = 2.5 (95%CI = 1.1–5.7) Dieldrin exposure was associated with elevated mortality among women with ER+ tumors	Suggests a link between PCB & dieldrin exposure and ER tumors progression
2002 Canada	[60]	Case control	314 women with breast cancer and 523 controls	Polychlorinated biphenyls	Serum 14 PCB (28, 52, 99, 101, 105, 118, 128, 138, 153, 156, 170, 180, 183 and 187)	At diagnosis; from 1994 to 1996	Association between PCB exposure and breast cancer risk	No association was found between PCB congeners and both tumor size and lymph node involvement	N/A
2003 USA	[61]	Cohort	224 women with nonmetastatic breast cancer	Organochlorines	Serum and adipose tissue 7 pesticides including DDE and DDT DDE trans-nonachlor, oxychlordane, β-HCH) 14 PCB (74, 99, 118, 138, 146, 153, 156, 167, 170, 172, 178, 180, 183, 187)	At diagnosis; from 1994 to 1996	Association between organochlorine concentrations and breast cancer recurrence	Total PCB levels were associated with an increased risk of breast cancer recurrence OR = 2.9, 95% CI = 1.02–8.2) (comparison between highest and lowest tertiles) Higher risk for PCB 118 OR = 4.0 (95% CI, 1.3–4.9) ORs for most PCB congeners were elevated, ORs > 2 for 118, 138, 153, 167, 183, and 187. Association DDT and DDE levels and breast cancer recurrence Statistically unstable elevated risk of recurrence associated with HCB, β-HCH, and trans-nonachlor	PCB, DDT and DDE exposure could be associated with cancer recurrence
2012 Brasil	[93]	Case control	81 women 38 controls, 9 benign tumors (fibroadenoma) and 34 breast cancer (both in situ and infiltrating ductal carcinomas) Total of 106 samples of breast tissue (34 from premonopausal patient) Medical record available for 57 patients	Trace element	Breast tissue total amount of Iron (Fe), Copper (Cu), Calcium (Ca) and Zinc (Zn) + Amount 2 cm away from the tumor (benign and malignant tumor)	At diagnosis; from 2003 to 2006	Overall survival	Patients with positive expression for Cu presented a poor overall survival (*p* < 0.001)	Exposure to copper could be associated with a decreased overall survival of breast cancer patients
2016 USA	[68]	Cohort	633 women with a first primary invasive or in situ breast cancer (66% postmenopausal)	Organochlorines	Serum DDT, DDE and chlordane	At diagnosis; from 1996 to 1997	Association between organochlorine concentration and overall and breast cancer-specific 5-year and 15-year mortality	−5-year overall mortality (comparison between highest and tertiles 2 and 3) DDT HR = 2.19 (95% CI = 1.02, 4.67) − 5-year breast cancer-specific mortality HR = 2.72 (95% CI = 1.04, 7.13) - At 15 years, concentrations of DDT and chlordane were modestly associated with overall and breast cancer-specific mortality (HR = 1.42 (95% CI =0.99, 2.06) and HR = 1.42 (95% CI = 0.94, 2.12) respectively)	DDT concentration was associated with all-cause and breast cancer-specific mortality
2016 USA	[63]	Cohort	627 women with a first primary invasive or in situ breast cancer	Polychlorinated biphenyls	Serum 22 PCB individually and by estrogenic group (101, 174, 177, 187, 199), antiestrogenic group (66, 74, 105, 118, 138, 170) and cytochrome P450 enzyme inducing group (99, 153, 180, 183, 203)	At diagnosis; from 1996 to 1997	Association between polychlorinated biphenyls concentration and overall and breast cancer-specific 5-year and 15-year mortality	−5-year overall mortality (comparison between highest and tertiles 2 and 3) PCB 174 HR = 2.22 (95% CI = 1.14–4.30) PCB177 HR = 2.12 (95% CI = 1.05–4.30) − 5-year breast cancer-specific mortality HR = 3.15 (95% CI = 1.23–8.09) −15-year breast cancer-specific mortality PCB 174 HR = 1.88 (95% CI = 1.05–3.36) PCB118 and antiestrogenic group inversely associated with overall mortality HR = 0.60 (95% CI = 0.39–0.83 and HR = 0.63 (95% CI = 0.43–0.92) respectively	See left column
2016 Spain	[62]	Cohort	103 women newly diagnosed with breast cancer (40.8% postmenopausal)	Organochlorines	Serum and adipose tissue p,p’-DDE, HCB PCB 138 PCB 153 PCB 180	From 2012 to 2014	Association between exposure to a group of organochlorines and tumor prognostic markers	HCB levels were associated with: -ER and PR expression (p-trends = 0.044 and 0.005, respectively) -decreased E-Cadherin expression (p-trends = 0.012) and p53 expression (p-trends = 0.027) PCB 180 adipose tissue levels were associated with HER2 expression (p-trend = 0.036) PCB 138 Serum were associated with - ER and PR expression (p-trends = 0.052 and 0.042, respectively)	Exposure to certain persistent organic pollutants (HCB, PCB 138 & 180) might be related to breast cancer aggressiveness
2017 Ukraine	[92]	Case control	40 samples of breast cancer tissue and 20 samples of intact breast tissue	Toxic metals	Breast tissue total amount of Iron (Fe), Copper (Cu), Zinc (Zn), Lead (Pb), Chromium (Cr) and Nickel (Ni)	At diagnosis	Link between chemical composition of neoplasic breast tissue and DNA methylation level, O6-methyguanine-DNA methyltransferase levels and prognostic-important receptors expression	The growth of toxic metals in neoplastic tissue is accompanied with: ↑ HER2/neu (*r* = 0.36, *p* < 0.05) ↑ p53 (*r* = 0.53, *p* < 0.01) ↑ Ki-67 (*r* = 0.47, *p* < 0.01) ↑ O6-methyguanine-DNA methyltransferase (*r* = 0.34, *p* < 0.05) ↓ ER (*r* = − 0.71, *p* < 0.01) ↓ PR (*r* = − 0.66, *p* < 0.01) ↑ pathological DNA methylation in tumor tissue (*r* = 0.41, *p* < 0.01)	Several toxic metals could stimulate the progression of breast cancer and reduce its sensitivity to treatment
2019 USA	[70]	Case control	456 white and 292 black women with breast cancer	Organochlorines	Serum lipid-standardized DDT and DDE levels	4.1 months after diagnosis; patient included from 1993 to 1996	Breast cancer-specific 5-year mortality Overall 20-year mortality conditional on 5-year survival	−20-year conditional overall mortality 1/ DDE: HR =1.95 (95% CI = 1.31–2.92) (comparison between highest versus lowest DDE tertile) 2/DDT: HR = 1.64 (95% CI = 1.10–2.44) (comparison highest vs undetectable DDT quantile) - 20-year conditional breast cancer-specific mortality 1/DDE: comparison levels above versus below the median) *Among women overall:* HR = 1.69 (95% CI = 1.06–2.68) *Among black women:* HRs =2.36 (95% CI = 1.03–5.42) *Among white women:* OR = 1.57 (95% CI = 0.86–2.89) (PInteraction = 0.42). *Among women with ER- tumors:* OR = 3.24 (95% CI = 1.38–7.58) *Among women with ER+ tumors:* OR = 1.29 (95% CI = 0.73–2.28) (PInteraction = 0.03).	Exposure to DDE/DDT may impact overall and breast cancer-specific survival
2019 France	[56]	Case control	91 women newly diagnosed with breast cancer 53 nonmetastatic and 38 metastatic (lymph node involvement)	49 persistent organic pollutants including PCB, PBDE, PBB and HBCD	Serum and adipose peritumoral tissue	At diagnosis; 2013–2017	Association between pollutants concentrations and lymph node involvement	2.3.7.8-TCDD concentrations in adipose tissue are associated with the risk of lymph node metastasis in patients with BMIs equal or higher than 25 kg/m2 OR = 4.48 (95%CI = 1.32–20.71) 2.3.7.8-TCDD concentrations and PCB 77 and 169 in adipose tissue are associated with risk of lymph node metastasis and tumor size	2.3.7.8-TCDD could contribute to the development of tumor metastasis in overweight patients

### Effect of chemicals on mechanisms of invasion and metastasis in breast cancer

#### Persistent Organic Pollutants

##### Dioxin

2.3.7.8-TCDD (named TCDD) is one of the most potent carcinogens ever tested and it is the most active congener within the group of AHR agonists. A recent epidemiological study of BC patient showed that TCDD concentrations in adipose tissue are associated with the risk of lymph node metastasis, especially in patients with BMIs equal or higher than 25 kg/m^2^ (OR = 4.48, 95%CI = 1.32–20.71) [56]. As described previously, the role of AHR in cancer progression and metastasis is complex and still controversial. Although dioxin is clearly a potent tumor promotor, a protective effect for BC progression has been suggested. It has been shown that the effects of AHR ligands on tumor growth are related to the ability of the receptor to antagonize ERα signaling. In both in vitro and in vivo models, dioxin was found to completely reverse the proliferative effects of estrogens [11, 12]. Other prior studies demonstrated that dioxin can disrupt the CXCL12/CXCR4 axis which has been shown to limit the metastasis of BC cells to the lung in mice. TCDD down-regulated both the G-protein-coupled receptor CXCR4 and its unique chemokine ligand, CXCL12 in MCF7 cells and decreased cell migration toward a CXCL12 gradient [13]. TCDD also suppressed proliferation, invasiveness and colony formation in vitro through the AHR signaling pathway, regardless of ER status and it promoted differentiation of a BC stem cell line [10]. In vivo, TCDD was found to suppress metastasis by approximately 50%, in a xenograft model with no effect on cancer cell proliferation or tumor growth [14, 23]. In another study which investigated the effects of a single, non-toxic dose of dioxin on the development of mammary tumors in female Sprague-Dawley rats treated with an oral dose of 7,12-dimethylbenzanthracene (a tumor initiator), it was found that the tumor volume in the controls was increased 3.9-fold after 21 days. In contrast, in a second group of rats with mammary tumors treated with dioxin, the mean tumor volumes decreased from 89.7 ± 53 mm^3^ to 24.9 ± 28.5 mm^3^ [9]. However, in contrast to these anti-tumorigenic data, other studies showed that dioxin induced an epithelial-mesenchymal transition (EMT), increased migration [15, 16] and induced mitochondrial dysfunction, stress signaling, and tumor invasion both in vivo and in vitro*.* The mechanism, similar to that described for mtDNA-depleted cells, directly targeted mitochondrial transcription and induction of mitochondrial stress signaling [17]. These paradoxical results may reflect differences in the tumor cell lines or assays. However, numerous other parameters could influence the behavior of the cells including the doses and the kinetics of treatments of the cell cultures or the composition of the cell culture medium (and, for example, the levels of competitive tryptophan-derived endogenous AHR ligands). Further mechanistic studies are warranted to understand the pro- or anti-tumor effects of AHR agonists and antagonists and the contextual role of the AHR in these processes.

##### Polychlorinated biphenyls (PCBs)

PCBs are a family of aromatic compounds which were used in industrial applications and electrical equipment until the early 1980s when they were banned in most countries because of human health concerns. However, due to their environmental persistence (as for POPs), the general population may be exposed to PCBs via a variety of routes and sources, including diet, ambient air, occupational settings and consumer products. It has been suggested that PCBs (105 and 118) contribute to high-grade BC tumors and overall poor prognosis in BC patients [57]. Seven epidemiological studies, between 2000 and 2019, investigated a potential link between PCB concentrations and BC prognostics factors or BC recurrence/survival. At diagnosis, the concentrations of PCBs were measured in serum and, in three studies, in breast adipose tissue [56, 58–63]. Demers et al. found that the concentration of PCB 153 was associated with an increased risk of lymph node involvement (OR = 2.12 95% CI = 1.5–4.3) in a first study [58]. However, this result was not confirmed in the largest study published by the same authors 2 years later (314 cases and 523 controls) [60]. The total amount of 14 PCBs was associated with an increased risk of BC recurrence in another large case control study (OR = 2.9, 95%CI = 1.02–8.2) [61]. With respect to BC survival, the total amount of 27 PCBs was associated with a risk for death, especially among patients with ER positive tumors [59, 63]. In vitro, PCBs enhance migration of MCF7 and MDA-MB-231 cells and tumor growth and the development of bone, lung and liver metastasis in mice by activating Rho-associated kinase (ROCK) [19]. Furthermore, an increasing amount of evidence indicates that the direct adhesive interaction between cancer cells and endothelial cells is a critical step in metastasis formation. It has been shown that PCBs can activate vascular endothelial cells which results in the disruption of the endothelial barrier function [64, 65]. PCBs induce proinflammatory reactions in human vascular endothelial cells by increasing oxidative stress. This is the result of an upregulation of the expression of genes encoding for monocyte chemoattractant protein-1 (MCP1), E-SELECTIN, intracellular adhesion molecule-1 (ICAM-1) and vascular endothelial cell adhesion molecule-1 (VCAM-1). These factors have been shown to play an important role in brain metastasis formation [66]. In particular, in BC, the exposure of the HMEC-1- (1human microvascular endothelial cell 1) cell line to PCB 104 induced endothelial hyperpermeability and markedly increased trans-endothelial migration of MDA-MB-231 cells [20]. These effects were associated with overexpression of vascular endothelial growth factor (VEGF). PCB 104-mediated elevation of VEGF expression was mediated by phosphatidylinositol 3-kinase (PI3K) but not affected by co-treatments with antioxidants or the NF-kappaB inhibitor. In addition, the PI3K-dependent pathway was involved in PCB 104-induced activation of activator protein-1, a transcription factor implicated in the regulation of VEGF gene expression. The VEGF receptor (KDR/Flk-1) antagonist SU1498 and the PI3K inhibitor LY294002 inhibited PCB 104-induced endothelial hyperpermeability. The authors concluded that PCB 104 may contribute to tumor metastasis by inducing VEGF overexpression which, in turn, stimulates endothelial hyperpermeability and trans-endothelial migration of cancer cells. The same authors used small interfering RNA and pharmacologic inhibitors to show that PCB 104 can activate epidermal growth factor receptor (EGFR) and Janus kinase 3 (JAK3) in a closely coordinated and cross-dependent fashion [21]. In this mechanistic study, activated EGFR and JAK3 stimulated, in concert, c-Jun NH(2)-terminal kinase (JNK) and extracellular signal-regulated kinase ½ (ERK1/2). They, further, increased the DNA-binding activity of transcription factors activator protein-1 and polyomavirus enhancer activator protein 3 which leads the transcriptional up-regulation of MMP-3 expression. These results indicate that the interplay among EGFR, JAK3, and mitogen-activated protein kinases may play an important role in PCB-induced MMP-3 expression and accelerated trans-endothelial migration of tumor cells.

#### DDE/DDT and other Organochlorine pesticides

Organochlorine pesticides (OCPs) are synthetic organic compounds used worldwide as insecticides, herbicides, termiticides and fungicides for agricultural and residential use. While they are banned in the USA and Europe, they are still used in some countries and, due to their long persistence in the environment, they are still contaminating food and soils. Epidemiological studies suggest that OCPs including dichlorodiphenyldichloroethane (DDE) and dichlorodiphenyltrichloroethane (DDT) could influence relevant pathways involved in breast cancer progression and impact BC prognosis and survival [61, 63, 67–69]. For example, DDE exposure has been associated with a dose-related increased relative risk of exhibiting both lymph-node involvement and a larger tumor [58] and a very recent study found that higher OCP levels in blood were associated with worse overall survival [70]. Exposure to dieldrin (another organochlorine pesticide) also was associated with the risk of mortality in patients with ER-positive tumors [59]. The limited amount of experimental data available show that DDT supports hormone-dependent BC cell growth by disrupting the estrogen-androgen balance and opposing the androgen signaling pathway that inhibits growth in hormone-responsive breast cancer cells [23]. In vitro, the *o,p’*-DDT isomer (but not the p,p’-DDT), which does not bind to tumor ER, supports estrogen-responsive tumor growth in ovariectomized rats in a dose-dependent manner [22]. These findings indicate a possible association between OCPs and BC progression through a non-genomic ER signaling pathway.

Hexachlorobenzene (HCB) is a widespread organochlorine pesticide which was used as a fungicide until the 1970s and still is released in several industrial processes. HCB was found to induce both cell proliferation and the insulin-like growth factor I (IGF-I) signaling pathway in MCF7 cells [24]. It also activated the c-SRC/HER1/STAT5b and HER1/ERK1/2 signaling pathways and cell migration in an AhR-dependent manner in MDA-MB-231 cells [25]. The same authors further showed that HCB (at a concentration of 5 μM) enhanced MMP2/9 expression, secretion and activity associated with cell invasion. This effect, in MDA-MB-231 cells, was mediated by the non-genomic AhR pathway which involves c-Src activation and the HER1/EGFR pathway. In vivo, HCB (0.3 and 3 mg/kg b.w.) enhanced subcutaneous tumor growth in 2 murine models (the MDA-MB-231 xenograft and C4-HI syngeneic models) [26]. Consistent with what is observed in vitro, the pesticide activated the c-SRC, HER1, STAT5b, and ERK1/2 signaling pathways and increased the levels of MMP2 and MMP9 proteins. The authors also observed that HCB stimulated lung metastasis regardless of the tumor hormone-receptor status. TGF-β1 signaling and AHR/TGF-β1 crosstalk was investigated in MDA-MB-231 cells in another study. HCB reduced *AHR* mRNA expression through TGF-β1 signaling but enhanced TGF-β1 mRNA levels through AHR signaling [27]. TGF-β1 is a secreted growth factor which has been linked notably to EMT. In this study, HCB increased the amounts of TGF-β1 protein and activation as well as the phosphorylation of downstream effectors such as SMAD3, JNK and p38. The authors showed that low and high doses of HCB had differential effects on the levels of AHR protein, localization, and activation. At a high dose (5 μM), it induced AHR nuclear translocation and AHR-dependent CYP1A1 expression. Taken together, these findings suggest that c-SRC and AhR are involved in HCB-mediated activation of Smad3, a mediator of TGF-β1 signaling, in a pathway linked to EMT. Furthermore, HCB enhanced cell migration and invasion through the SMAD3, JNK, and p38 pathways whereas ERK1/2 was involved only in HCB-induced cell migration.

#### Consumer product chemicals

##### Bisphenol A

BPA is a xenoestrogen which is used in the manufacturing of polycarbonate plastics and epoxy resins. The major source of exposure for the general population is food packaging. Because of its widespread presence in the environment and its estrogenic activity both in vivo and in vitro, numerous studies have investigated the potential for adverse effects of BPA exposure on human health. They suggest that BPA is significantly correlated with diseases such as diabetes, cardiovascular diseases, increased inflammation and cancer. The role of BPA in BC progression and resistance to chemotherapy has been suggested in several studies.

Bisphenol A first was described as a pro-estrogenic compound. It has been shown that breast cancer cells exposed to BPA exhibit a gene expression profile which is characteristic of tumor aggressiveness and which is associated with poor clinical outcomes for breast cancer patients [33]. Studies conducted in the MCF-7 cell line estrogen receptor-positive cells (ER+) showed that low levels of BPA significantly increased proliferation [30] and induced MCF-7 breast cancer cell proliferation through upregulation of genes that promote the cell cycle and downregulation of anti-proliferative genes [31, 32]. However, BPA is a low-affinity ligand of estrogen receptors and it has been shown, at environmentally relevant doses, to antagonize the cytotoxicity of multiple chemotherapeutic agents (doxorubicin, cisplatin, and vinblastine) in both ER-alpha-positive and -negative breast cancer cells, independent of the classical ERs, by increasing expression of anti-apoptotic proteins. It was shown that both cell types express alternative ERs, including G-protein-coupled receptor 30 (GPR30) and members of the estrogen-related receptor family [71]. The involvement of these alternative ER targets was confirmed by Castillo Sanchez et al. who showed that BPA promoted migration and invasion and increased the number of focal contacts in MDA-MB-231 breast cancer cells through a GPR-dependent pathway [34]. This pathway is controversial as Zhang et al. have found that nanomolar concentrations of BPA increased wound closure and invasion of BC cell lines MDA-MB-231 and BT-549 through increased expression matrix metalloproteinases 2 (MMP2) and MMP9 protein and mRNA levels, although it displayed no effect on the expression of vimentin and fibronectin in triple negative breast cancer (TNBC) cells. The expression of GPR30, which has been suggested to mediate rapid estrogenic signals, was not modified in BPA-treated MDA-MB-231 and BT-549 cells. Its inhibitor, G15, also had no effect on BPA-induced MMP expression or cell invasion. This suggests that GPR30 is not involved in all BPA effects [29]. However, the BPA effects may be mediated through its high-affinity nuclear receptor, the estrogen-related receptor γ (ERRγ) (but not ERRα or ERRβ). Indeed, the knock-down of ERRγ markedly attenuated BPA-induced expression of MMP-2 and MMP-9 [28]. Further, BPA treatment activated both ERK1/2 and AKT in TNBC cells. Inhibitors of both ERK1/2 and AKT attenuated BPA-induced ERRγ expression and cell invasion of MDA-MB-231 cells. Taken together, the results suggest that BPA increase the expression of MMPs and the in vitro motility of TNBC cells via ERRγ. Activation of ERK1/2 and AKT also participated in this process. To conclude, Castillo-Sanchez et al. found that BPA induced activation of FAK, SRC, and ERK2, three mediators which activate cell migration. BPA also induced an increase of AP-1- and NFκB-DNA binding activity through a SRC- and ERK2-dependent pathway [34].

##### Phthalates

Phthalates are one of the more abundant synthetic chemical contaminants. Phthalates, which include butyl benzyl phthalate (BBP), di(n-butyl) phthalate (DBP), and di (2-ethylhexyl) phthalate (DEHP) are used as plasticizers and also are widely used in food wraps and cosmetic formulations. They are suspected to be endocrine-disrupting chemicals and have been shown to increase cell proliferation [39], tumor mobility, and invasiveness of tumor cells [19, 64–66, 70, 72]. Phthalates also are suspected to cause the proliferation and metastasis of BC cells and tumor progression via up-regulating histone deacetylase 6 (HDAC6) [36]. In the later study, phthalate-induced HDAC6 expression was mediated by the ER-alpha/EGFR/PKA/AP-2a pathway and led to vimentin expression which involved the AKT, GSK3β, and β-catenin signaling cascade. The authors also showed that the effects of DBP and BBP on HDAC6 gene expression were mediated through the nongenomic pathway of the AHR in ER-negative breast cancer cells (MDA-MB-231). They found that phthalates stimulated the AHR located at the proximity of the cell membrane and triggered the downstream cyclic AMP (cAMP)-PKA-CREB1 signaling cascade [37]. Phthalates also activated peroxisome proliferator activated receptors (PPARs) which may eventually lead to high proliferation of MCF7 cells [38]. Finally, phthalates were shown to affect the sensitivity to tamoxifen, which suggests a role in chemotherapeutic drug resistance. They can inhibit tamoxifen-induced apoptosis in ER-positive MCF-7 cells (but not in ER-negative cells) [40].

##### Benzophenone / Nonylphenol

Benzophenone-1 (2,4-dihydroxybenzophenone, BP1) and nonylphenol (NP), which are discharged from numerous industrial products (lubricating oil additives, laundry and dish detergents, emulsifiers …), are known EDC which are defined as environmental compounds that produce human adverse effects by disrupting the endocrine system. A study of the effects on proliferation and metastasis of MCF-7 human BC cells expressing estrogen receptors (ER) [41] found that treatment with BP1 (10^− 5^–10^− 7^ M) and NP (10^− 6^–10^− 7^ M) promoted proliferation of MCF-7 cells similarly to the positive control 17-beta-estradiol (E2). This response was abrogated in presence of the ER antagonist ICI-182,780. Moreover, addition of BP1 or NP markedly induced migration of MCF-7 cells similar to E2. Alterations in transcriptional and translational levels of proliferation and metastasis-related markers included an increase in the expression of cyclin D1, a cell cycle progressor, and cathepsin D and a decrease in the expression of p21, a negative regulator of cell cycle progression at G1 phase. The BP1- or NP-induced alterations of these genes were blocked by ICI-182,780 which suggests that changes in the expression of these genes may be regulated by an ERα-dependent pathway. Authors concluded that BP1 and NP may accelerate growth of MCF-7 breast cancer cells by regulating cell cycle-related genes and promote cancer metastasis through amplification of cathepsin D. Similar results concerning the effects of BP1 were found in BG1 human ovarian cancer cells which express the ER with the up-regulation of cyclin D1 [73]. In xenograft mouse models transplanted with BG1 cells, 8 weeks treatment with BP1 or E2 significantly increased tumor mass formation as compared to a vehicle. Histopathological analysis of the tumor sections of the E2 and BP1 groups showed extensive cell formations with high densities and disordered arrangements. These were accompanied by an increased number of BrdU-positive nuclei and the over-expression of cyclin D1 protein. Taken together, these results suggest that BP1 exerts xenoestrogenic effects by stimulating the proliferation of ER-positive cancer cells via the ER signaling pathway.

##### Per and Polyfluoroalkyl substances (PFOS/ PFOA)

Perfluoroalkyl acids (PFAAs) are prominent environmental toxicants which are used in industrial and consumer products because of their stain-resistant and water-repellant characteristics. The two most widely known PFAAs are perfluorooctanoic acid (PFOA), and perfluorooctane sulfate (PFOS) [74]. They are suspected to be endocrine disruptors and potentially linked with BC but the mechanisms underlying their actions are unknown. We found no references that specifically investigate the role of PFAAs in cancer progression. However, there are some data which suggest their role in late stage of cancer development [42, 43]. A tumorigenic activity of PFOA (50–100 μM) and PFOS (1–10 μM) was found in MCF10 human breast epithelial cells. These cells exhibited a higher growth rate as compared to controls. PFOA promoted MCF-10A proliferation by accelerating the transition from G0/G1 to S in the cell cycle due to increased cyclin D1 and CDK4/6 levels and a concomitant decrease in p27. PFOS had the same effect on the cell cycle due to increased levels of CDK4 and decreased amounts of p27, p21, and p53. The authors also showed that PFOA and PFOS are able to stimulate cell migration and invasion which underlies their role in BC progression. Moreover, the ER antagonist ICI 182,780 had no effect on PFOA-induced cell proliferation, whereas the PPARα antagonist GW 6471 was able to prevent the MCF-10A proliferation. These results suggest that the underlying mechanisms involve PPARα (a target suspected to relay PFOS and PFOA effects in other organs).

#### Food preparation

Genotoxic carcinogens potentially are present in the human diet. Two important examples are benzo(a) pyrene (BaP) and 2-amino-1-methyl-6-phenylimidazo [4,5-b] pyridine (PhIP) which are by-products of food processing and preparation. BaP is a polycyclic aromatic hydrocarbon generated by incomplete combustion of organic substances which, thus, contaminates numerous foodstuffs. PhIP is a heterocyclic amine which is formed when meat is cooked. Both are also found in cigarette smoke.

##### Benzo(a)pyrene

BaP is a mammary carcinogen in rodents and it contributes to the development of human breast cancer. It significantly increased invasion in MDA-MB-231 cells. Treatment of MDA-MB-231 cells with Vomitoxin (a selective COX-II inducer) also enhanced invasion and co-treatment with NS398 (a selective COX-II inhibitor) attenuated BaP-induced invasion. These results suggest that COX-II is involved in the effects of BaP. The number of COX-II immunopositive cells and the levels of COX-II protein were increased in immunohistochemical staining and western blots, respectively, following exposure of cells to BaP. BaP exposure also induced a profound (46-fold) increase in PGE2 production by MDA-MB-231 cells. The AHR antagonists, resveratrol (RES) and alpha-naphthoflavone (alpha-NF) had no effect individually but combined treatment with RES and alpha-NF inhibited BaP-induced invasion. This suggests a role for the AHR signaling pathway [44]. Another study demonstrated that BaP induces cell migration through a lipoxygenase- and Src-dependent pathway as well as activating the focal adhesion kinase (FAK), SRC and the extracellular signal-regulated kinase 2 in MDA-MB-231 cells but not in the mammary nontumorigenic epithelial cells MCF12A. This suggests that BaP plays a role in BC invasion and the metastasis process and not in BC initiation [35]. The authors also showed that BaP promotes an increase of αvβ3 integrin-cell surface levels and an increase in secretion of metalloproteinase (MMP)-2 and MMP-9. In another study, Guo et al. showed that the pro-oxidant properties of BaP (enhanced ROS production) stimulated the ERK signaling pathway which activates the expression and activity of MMP in MCF7 and MDA-MB-231 cells. As a consequence, cell migration and invasion were enhanced [45]. Cumulative BaP exposure leads to increased tumor growth and liver and lung metastasis in a mouse model. Taken together, these data suggest that BaP can act on several steps of the metastatic cascade and participate in BC progression.

##### 2-Amino-1-Methyl-6-Phenylimidazo [4,5-B]pyridine

2-amino-1-methyl-6-phenylimidazo [4,5-b] pyridine (PhIP) is a genotoxic, cooked meat-derived which induces cancers of the colon, prostate and mammary gland when fed to rats. The carcinogenic effects of cumulative exposures to PhIP at physiologically achievable, pico to nanomolar concentrations also has been demonstrated in vitro using human breast epithelial MCF10A cells [47]. In this study, progressive carcinogenesis was evidenced by increasingly acquired cancer-associated properties which included reduced dependence on growth factors, anchorage-independent growth, acinar-conformational disruption, proliferation, migration, invasion, tumorigenicity with metastasis and increased stem-like cell populations. These biological changes were accompanied by biochemical and molecular changes such as upregulated *H-RAS* gene expression, extracellular signal-regulated kinase (ERK) pathway activation, *NOX-1* expression, reactive oxygen species (ROS) elevation, increased HIF-1α, SP1, tumor necrosis factor-α, matrix metalloproteinase (MMP-2, MMP-9), aldehyde dehydrogenase activity and reduced expression of E-cadherin. Another study showed that treatment of MCF7 and T47D with sub-nanomolar concentrations of PhIP enhanced invasion and migration in a dose-dependent manner [46]. These effects were negated by the antiestrogen ICI 182,780. The PhIP-induced invasive phenotype was associated with an enhanced expression of cathepsin D and cyclooxygenase-2 and increased activity of matrix metalloproteinase 9 (MMP9).

#### Other polycyclic aromatic carbons

##### Cigarette smoke

Cigarette smoke has an extremely complex chemical composition and contains numerous toxic and carcinogenic substances which include many polycyclic aromatic hydrocarbons (PAHs). Multiple epidemiological studies have established the association between active and involuntary exposure to cigarette smoke and an increased risk of BC. Although little is known about the effects of tobacco carcinogens on breast cancer progression and metastasis. Several epidemiological studies have shown that women who smoke have a higher rate of fatal BC than nonsmoking women [75–78]. Two initial works demonstrated an association between smoking and pulmonary metastasis among women with BC [79, 80]. A prospective, randomized animal study showed that cigarette smoke exposure was associated with an increase in the total pulmonary metastatic burden in a murine model of metastatic mammary cell cancer [48]. Experimental animals were exposed to cigarette smoke at different concentrations chosen to approximate active cigarette smoking in specialized exposure chambers. One week after the initiation of exposures, mouse mammary tumor cells were injected into the tail veins of the animals and pulmonary metastases were counted and measured 3 weeks later. The mean metastatic burden in the lungs was consistently greater for the smoke-exposed animals. However, the molecular mechanisms were poorly investigated. In a chronic (several weeks) cigarette smoke exposure model, both non-tumorigenic (MCF10A, MCF12A) and tumorigenic (MCF7) breast epithelial cells acquired mesenchymal properties such as a “fibroblastoid” morphology, increased anchorage-independent growth and increased motility and invasiveness [49]. Analysis by flow cytometry showed that treatment with cigarette smoke extract leads to the emergence of a CD44(hi)/CD24(low) population in MCF10A cells and of a CD44+/CD49f + population in MCF7 cells. These changes indicate that cigarette smoke causes the emergence of cell populations which bear the markers of self-renewing stem-like cells with increased colony formation. In vivo, cigarette smoke extract increased the survival of MCF10 cells as well as their ability to colonize the mammary ducts. In contrast, MCF7 cells were more prone to metastasize from a subcutaneous injection site independent of cigarette smoke effects on the host and stromal environment. These phenotypic modifications were associated with gene expression changes characteristic of EMT.

Several studies have attempted to identify which compounds of cigarette smoke, among the more than one hundred potential carcinogens, could lead to enhanced BC metastasis. Cigarette smoke contains ligands of the AHR (including BaP) but also nicotine, the major addictive component of cigarettes. Evidence from in vitro studies employing cell cultures, in vivo studies on rodents as well as studies on humans, inclusive of epidemiological studies, indicate that nicotine by itself, independent of other tobacco constituents, may stimulate a number of effects which are of importance for cancer development [81–83]. Nicotine also has been found to induce cell proliferation and angiogenesis, to induce morphological changes characteristic of a migratory, invasive phenotype and to confer resistance to apoptosis in lung cancer cell lines through the nicotinic acetylcholine receptors (nAChRs) [50]. These pro-invasive effects of nicotine were mediated by alpha7-nAChRs in non-small cell lung cancer. RT-PCR analysis showed that the alpha7-nAChRs also were expressed on human BC. Further, nicotine was found to promote proliferation and invasion in 2 human breast cancer cell lines (MCF7, MDA-MB-468) through a nAChR, SRC and calcium-dependent signaling pathway. Similarly, in this study, nicotine was found to induce changes in gene expression consistent with EMT. Another recent study confirmed that nicotine-treated MCF7 cells exhibited changes in cell structure, cellular motility (related to the relocation of F-actin) and an enhanced MCF7 CD44 + CD24- cancer stem cell population [51]. The authors also demonstrated chemoresistance effects induced by nicotine (towards doxorubicin, a chemotherapy routinely used in BC).

##### 7,12-Dimethylbenz(a)Anthracene

7,12-Dimethylbenz [a] anthracene (DMBA) is a widely studied polycyclic aromatic hydrocarbon which has long been recognized as a very potent carcinogen. It has been shown that DMBA can promote a more invasive, mesenchymal phenotype in the Rel-3983 cell line, a cell line established from a mouse mammary tumor virus (MMTV)-c-*rel* transgenic mouse model constructed to test, directly, the role of the nuclear c-Rel subfamily of NF-κB proteins in breast cancer [52]. Indeed, NF-κB was found to be constitutively activated in BC cells and large number of nuclear c-Rel were found in a large several primary human BC. The authors showed that DMBA-treated cells displayed an increased rate of proliferation, displayed growth to a higher cell density, acquired the ability to grow in soft agar and matrigel and showed a loss of E-cadherin expression. As compared to control cells, DMBA-treated cells displayed increased NF-kappaB binding and increased amounts of the NF-kappaB transactivating subunits c-REL, RELA, and RELB which appeared to be functional as judged by the concomitant induction of c-MYC and vimentin, two NF-kappaB target genes. In this mechanistic study, ectopic expression of a super repressor mutant of IkappaB-alpha reduced cell growth and invasive morphology in Matrigel which confirmed the role of NF-kappaB in the epithelial to mesenchymal transition.

#### Alcohol

Accumulating evidence indicates that exposure to alcohol may enhance the progression and aggressiveness of existing mammary tumors via multiple potential mechanisms [53]. Alcohol may increase the mobility of cancer cells by inducing cytoskeleton reorganization and by enhancing cancer cell invasion through degradation and reconstruction of the extracellular matrix [84–87]. It promoted EMT [88], impaired endothelial integrity, thereby increasing the dissemination of breast cancer cells and facilitating metastasis in animals [89]. Alcohol may also stimulate tumor angiogenesis through the activation of cytokines and chemokines which promotes tumor growth [90, 91]. Additionally, alcohol may increase the cancer stem cell population which affects neoplastic cell behavior, aggressiveness and the therapeutic response [89]. Alcohol can be metabolized in mammary tissues and breast cancer cells which produce reactive oxygen species (ROS) which causes oxidative stress. Several studies found that the epidermal growth factor receptor (EGFR) family, particularly ERBB2 (a member of this family), was involved in alcohol-mediated tumor promotion. For example, breast cancer cells or mammary epithelial cells over-expressing ERBB2 were more sensitive to the tumor promoting effects of alcohol which suggests that oxidative stress and EGFR/ ERBB2 signaling play an important role in this process [53].

#### Toxic metals

Increased amounts of toxic metals (Iron, Copper, Zinc, Lead, Chromium, Nickel) in breast malignant tumors (as measured by atomic absorption spectrophotometry and energy-dispersion spectrometer) have been shown to be associated with poor molecular prognostic factors [92]. Tumor growth induced by toxic metals was accompanied by an increase in expression of HER2/neu, p53, Ki-67, 06-methylguanine-DNA methyltranferase and a decrease of ER-alpha and PR expression. The same authors also showed that the increment of pathological DNA methylation was accompanied by the accumulation of toxic metals in tumor tissues. They concluded that toxic metals stimulated the progression of breast cancer and reduced its sensitivity to treatment. Higher concentrations of Ca, Fe, Cu, and Zn trace elements (TE) were found in neoplastic breast tissues (malignant and benign) as compared to normal tissues [93]. In this study, the expressions of all TE were found to be statistically correlated with well-known prognostic factors for breast cancer. There was also a statistical correlation between copper and overall survival which suggested that copped could be used as a biomarker. Long-term cadmium exposure has been shown to promote migration and invasion of BC cells through the TGIF/MMP2 signaling axis [94]. Tungsten was found to target the tumor microenvironment and to enhance breast cancer metastasis [55]. In a BC- xenograft model, tungsten slightly delayed primary tumor growth but it significantly enhanced lung metastasis. In vitro, tungsten did not enhance BC cell proliferation or invasion. These data suggest that tungsten was not acting directly on BC primary tumor cells to enhance invasion. In contrast, tungsten changed the tumor microenvironment which enhanced parameters known to be important for cell invasion and metastasis (such as activated fibroblasts, matrix metalloproteinases (MMP9), and myeloid-derived suppressor cells) and which were associated with a poor prognosis in humans. Furthermore, toxic metals were able to cause various epigenetic dysregulations which are believed to play important roles in their carcinogenicity. However, the mechanisms are largely unknown. A very recent review concerning metal carcinogens (arsenic, cadmium, nickel and hexavalent chromium) suggested that metals could produce CSC-like cells through dysregulated epigenetic mechanisms [54].

## Discussion

### Scientific evidence from epidemiological studies

A summary of the studies in humans which investigated exposure to environmental chemical disruptors and BC aggressivity, or survival is given in Table 2. Organochlorine pesticides (OCPs) PCB are the chemicals which were studied the most frequently. Several studies suggested that exposure to DDT and DDE (two OCPs) may impact BC aggressivity and overall and BC survival [58, 61, 68]. Exposure to dieldrin an estrogenic organochlorine OC, also may affect BC mortality among patients with ER- positive tumors [59, 69]. Total PCB levels have been associated with an increased risk of BC recurrence [61], risk of death among women with ER-positive tumors [59] and overall survival [63] but no association has been found between PCB congeners and tumor size or lymph node involvement [60]. Some of these studies are of particular interest because the shorter time interval between exposure and outcome increases confidence in the validity of the exposure measurement and suggests that ongoing exposures may have health implications [59–61]. Studies of disease progression also have an advantage in that biological measurements are made near diagnosis and, thus, are more indicative of exposure during the time relevant to the outcome studied. However, methodological limits include the heterogeneity in research designs in uncontrolled studies, the lack of information about premenopausal status or treatment data and the lack of preclinical markers identify associations which may be obscured by disease latency. Further epidemiological studies using rigorous methodology are warranted.

### Critical steps in cancer progression and chemoresistance targeted by environmental chemicals

The formation of metastases constitutes a complex process of molecular and biochemical events which are performed by multiple actors and which is called the “invasion-metastasis cascade”. The metastatic cascade requires that tumor cells first detach from the primary tumor, which is allowed by the loss of epithelial proteins, then invade through extracellular matrix, intravasate into the bloodstream and/or the lymphatic circulation, migrate to a target site with specific adhesion properties, attach to target endothelium, extravasate and invade into the target tissue and, finally, establish tumor growth including vascularization [95]. Among the mechanisms involved in cancer metastasis, two critical pathways were found to be linked, particularly, to the exposure to low-dose doses of environmental pollutants and resistance to anticancer drugs in this review: the epithelial-to-mesenchymal transition (EMT) and the emergence and progression of cancer stem cells (CSCs).

#### Epithelial-to-mesenchymal transition

EMT is a biological process by which differentiated epithelial cells lose their epithelial cell characteristics and acquire a migratory, mesenchymal phenotype. This occurs during normal embryonic development or tissue regeneration. However, this process also is involved in tumor progression and cancer cell invasion and metastasis. More specifically, the generation of tumor cells is accompanied by the acquirement of stem cell properties which, subsequently, play a major role in resistance to cancer treatment [96–98]. Cancer cells undergoing EMT undergo important morphological changes which are induced by signal transduction pathways that reduce E-cadherin expression (one of the major cell adhesion proteins), drive the disassembly of intercellular adhesion complexes and promote actin stress fiber and focal adhesion formation. These types of cellular changes result in a phenotypic transition to an elongated, mesenchymal cell that expresses extracellular matrix-remodeling enzymes and which has an increased capacity for migration and invasion. Loss of expression of E-cadherin, one of the most frequently used markers for EMT, is associated with a more invasive phenotype in many types of human carcinomas [99, 100]. We showed, in a previous work, that 2,3,7,8-Tetrachlorodibenzodioxin (TCDD), the most frequently studied and the most toxic of all dioxins, decreased E-cadherin expression and favored EMT and cell migration in vitro through the activation of the aryl hydrocarbon receptor (AHR) [16, 101]. Several other studies which have used a variety of cancer cell lines found that EMT could be triggered by exposure (sometimes even to low-doses) to various environmental chemicals such as benzo(a) pyrene (BaP), a polycyclic aromatic hydrocarbon (PAH), an AhR agonist, produced by incomplete combustion of organic components (EDC) [102], nicotine [50, 103, 104], toxic metals such as cadmium and chromium [105, 106], pesticides [107] or other endocrine-disrupting chemicals [5]. The literature is prolific and shows that environmental and synthetic chemicals have the potential to induce cancer metastasis through regulation of EMT markers and activation of migratory processes. These toxic chemicals are able to regulate many transcription factors (SNAIL, SLUG, ZEB1, ZEB2, and TWIST) and several signaling pathways mediated by transforming tumor growth factors ß (TGFß), the WNT/ß-catenin, NOTCH, HEDGEHOG, NF-kappaB pathways and the tyrosine kinases receptor. Several of these factors have been shown to be crucial for the regulation of self-renewal and the maintenance of CSCs [108].

#### Cancer stem cells emergence and progression

Cancer stem cells are a class of pluripotent cells that have been observed in most solid and hematologic cancers. CSCs constitute a small percentage (0.05–1%) of tumor cells within the tumor mass. They express stem cell marker genes such as OCT4 (octamer-binding transcription factor 4), SOX2 ((sex determining region Y)-box 2), Nanog, c-kit (tyrosine-protein kinase Kit), ABCG2 (ATP-binding cassette super-family G member 2), and ALDH (aldehyde dehydrogenase). These cells possess a capacity for self-renewal that makes them immortal and it is now clear that they are involved in tumor development, cell proliferation, metastatic dissemination and resistance to chemotherapy and radiotherapy [109–111]. Accumulating evidence has suggested that it is CSCs that exhibit invasive properties, support the formation of metastasis and contribute to cancer recurrence. The identification of CSCs, and their recognition as major actors in cancer progression through their invasive properties, has constituted a landmark discovery in cancer research and, specifically, in cancer therapy. Multiple, promising CSC-targeting drugs are progressing in preclinical and clinical studies [112].

The involvement of chronic exposure to current background levels of carcinogens in the regulation of self-renewal and maintenance of CSCs has been suggested by several studies. Such molecules act on several cellular processes which include the emergence and proliferation of CSCs. Normal breast tissue is composed of epithelial cells, fibroblasts, adipocytes, blood vessels and stem cells (which can renew the tissue through aging). The homeostasis of this tissue is maintained by the balance of several cytokines including Bone Morphogenetic Proteins (BMP) 2 and 4, which act, respectively, on the proliferation and the differentiation of breast luminal cells. BMPs have been implicated in late stages of tumorigenesis and metastasis. Chapellier et al. showed that bisphenol A (BPA) and bisphenol S (BPS) were able to shift the balance of secreted BMP molecules in favor of BMP2 which promotes malignant transformation and which regulates the emergence of a niche of mammary stem/progenitor cells with features similar to CSCs [113, 114]. Cadmium significantly induced the expression of CSC gene markers in breast and liver cancer cell lineages and it promoted the conversion of non-CSCs to CSCs in the MCF-7 and HepG2 cell lines [115]. EDCs also were able to stimulate CSC proliferation. Benzyl butyl phthalate (BBP), a toxic phthalate used as plasticizer in diverse products, has been shown to promote breast CSCs through the activation of the AhR and the stimulation of sphingosine kinase 1 (SPHK1)/sphingosine 1-phosphate (SP1)/sphingosine-1-phosphate receptor 3 (S1PR3) signaling. In a mouse BC xenograft model, BBP increased the incidence of lung metastasis and this effect was reversed with SPHK1 or S1PR3 knockdown tumor cells [116]. A link between CSCs and environmental chemical exposure also has been suggested in a variety of studies in other cell lines or cancer types. Arsenic, a class I carcinogen, triggered the proliferative potential of epidermal keratinocytes and decreased their exit from the germinative compartment under conditions that promoted differentiation of untreated cells [117]. Exposure to low doses of arsenic (over 18 weeks) was reported to transform human prostate epithelial stem/progenitor cells, WPE, into CSCs, a process linked to the downregulation of the tumor suppressor, phosphatase and tensin homolog expression (PTEN) [118–121].

CD34-positive cells isolated from a human skin keratinocyte line and exposed to arsenic appeared to contain proportionally more putative CSCs than normal stem cells isolated from control cells [122]. In addition, Hu et al. have shown that cultured prostaspheres which are composed of prostate stem/progenitor cells and which express estrogen receptors alpha and ß (ERα, ERß), and GPR30 (GPER1) proliferated in response to low-dose estradiol and BPA and increased the number of stem-like cells in the side populations [123].

Collectively, these data support the concept that exposure to environmental chemicals may promote cancer cells dedifferentiation to CSCs which have a crucial role in cancer progression, including BC. However, further studies are needed to definitively establish this link.

#### Link between environmental chemicals and chemoresistance

Resistance to chemotherapy is an important issue for treating the most commonly seen solid tumors. Although many studies have been devoted, in the past, to the role of pollutants in the initiation of cancer, and more recently in the invasion and metastasis process, to our knowledge only one recent paper has investigated the influence of environmental disruptors on the response of cancer patients to chemotherapy. In this study, the authors showed that BaP reversed the effect of cisplatin, 5-flurouracil, and paclitaxel in the WHCO1 esophageal cancer cell line by reducing drug-induced cell death and apoptosis by 30–40% as compared to cells treated by drugs alone [124]. The mechanism(s) by which cancer cells develop chemoresistance is a subject of debate. Many reports suggest cancer stem cells, EMT and the tumor microenvironment [4, 125–127]. To evaluate the effects of environmental chemicals on such mechanisms, we examined the literature for studies which linked these chemicals to cancer chemotherapy resistance. We found several papers which suggested an indispensable role of the EMT and of CSCs in cancer cell drug resistance. In 1992, Sommers et al. described the loss of epithelial markers and the acquisition of vimentin expression in Adriamycin- and Vinblastine-resistant human breast cancer cell lines and they hypothesized that EMT cells have advantages in growth capabilities as compared to non-EMT cells after drug treatment [128]. Since this first report, chemoresistance to different drugs such as oxaliplatin and paclitaxel often has been linked to the EMT and has been reported in several cell lines and animal models [129–134]. However, a consensus has not been reached on this matter [96, 135, 136] and more experimental studies are needed to further the debate. Conversely, the association between stem-cell properties and resistance to chemotherapy is well established. In BC, many signaling pathways in cells indirectly promote CSC-mediated chemoresistance which increases stemness properties and self-renewal of CSCs [39, 137–144]. The association between environmental chemicals and CSC emergence described above necessitate the exploration of direct links between pollutants and anti-cancer drug resistance.

#### Potential role of AhR in cancer progression and metastasis development

Many of the pollutants described above are AHR ligands. The AHR has been linked to the regulation of the EMT and the proliferation of CSC. However, historically, the AHR has been described as a xenobiotic-activated transcription factor which is involved in detoxication pathways [145]. It is activated, indeed, by many environmental pollutants such as dioxins, furans, polychlorinated biphenyls and polycyclic aromatic hydrocarbons protects organisms against chemical toxicity. In the last decade, AHR has been shown to play a critical role in many alternative pathways like autoimmunity, metabolic imbalance, inflammatory skin, gastro-intestinal disease among others. Thus, accumulating evidence indicates that the AHR may be involved in proliferation, cell migration and angiogenesis [16, 101, 145–150] and may play an important role in cancer progression in a variety of cancer types [151–162]. However, studies are controversial, and it is still unclear whether the AHR promotes or inhibits cancer aggressiveness in any given tumor type [163–170]. For example, a recent study showed that inhibition or knockdown/knockout of the AHR consistently reduced human cell invasion, migration, and metastasis in triple negative and inflammatory breast cancer cell lines (decrease in the invasion-associated genes Fibronectin, VCAM1, Thrombospondin, matrix metalloproteinase 1) and increased the expression of genes associated with decreased tumor progression (CDH1/E-cadherin) [18]. The authors found that 2.3.7.8-TCDD and 3,3′-diindolylmethane (another AHR agonist) inhibited irregular colony formation in Matrigel (correlated with stem-cellness) and blocked metastasis in vivo while accelerating cell migration. This example, among many others, demonstrates a complex role of the AhR in cancer progression and metastasis and shows that underlying mechanisms are still unclear and could depend on the nature of the ligand as well as on the cellular context such as the microenvironment. Moreover, numerous ligands can activate the receptor but with a different outcome. Our own studies showed that, according to the type of ligand, the AHR regulates different types of target genes. Finally, the presence of endogenous ligands (including tryptophan derivatives) probably interferes with the outcome of the signaling pathway. Thus, further investigations using relevant in vitro and in vivo models are warranted in order to elucidate the potential effect of the AHR in cancer spreading.

Overall, the entirely different outcomes which have been observed for this signaling pathway (or others such as toxic metals) deserve a better contextual characterization to properly understand which conditions are pro- or anti-metastatic. Further, environmentally relevant mixtures of pollutants might contain pro- and anti-metastatic compounds which suggests that the effects of pollutants on migratory and invasive pathways require a better characterization not only as a single molecules but also as relevant “cocktails”.

## Conclusion

Despite encouraging advances in both local and systemic treatment over the last few years, cancer remains a leading cause of death worldwide and metastasis is the major cause of cancer morbidity and mortality. The main outcome of this review was to summarize the main findings related to the role of environmental contaminants in the promotion of invasion and metastasis in BC and in chemoresistance. We report important links between numerous environmental pollutants and several cell functions implicated in breast cancer progression and metastasis. Studies of TCDD, the most active congener within the group of AhR agonists, are complex to analyze as experimental studies are controversial although a recent epidemiologic study associated its concentrations in the adipose tissue surrounding breast tumors with a more aggressive tumorigenic phenotype in overweight patients. Among POPs, coherent data were found between epidemiological and experimental studies which suggests that several PCBs are linked to BC aggressiveness. However, the most conclusive analyses at the epidemiological level were based on aggregates of PCBs and more extensive investigations need to be performed to identify which specific congeners are involved in BC metastasis. Three organochlorine pesticides (OCPs), DDT, DDE and dieldrin also were associated epidemiologically and experimentally to BC progression although through unexpected non-genomic mechanisms of action involving estrogen receptor. Diverse experimental data on HCB, another OCP, involved stimulation of cellular metastatic processes. Our review also provides strong evidence, mostly at the experimental level, that BC progression is linked to common non-persistent organic and inorganic compounds are (BPA, three phtalates, benzophenone-1, nonylphenol, benzo(a) pyrene and other PAHs, PhIP, alcohol, iron, copper, zinc, lead, chromium and nickel).

One part of the review was dedicated to PFOS and PFOA, the most famous perfluoroalkyl acids which are considered as POPs but which possess properties different from TCDD, PCBs or OCPs which are highly lipophilic and poorly metabolized by most organisms. Whereas the latter accumulate in adipose tissues or liver, PFOS and PFOA bioaccumulate in the blood compartment and in the liver. One experimental study showed that they can stimulate BC migration and invasion (ostensibly through the nuclear receptor, PPARα), however, additional experimental and epidemiological studies clearly are needed. In conclusion, although several pertinent pathways for the effects of xenobiotics have been identified, the mechanisms of actions for multiple other molecules remain to be established. The integral role of xenobiotics in the exposome needs to be further explored through additional relevant epidemiological studies that can be extended to molecular mechanisms. This is a major public issue considering the significant number of deaths due to advanced stages of breast cancer and other types of tumors.

## Supplementary Information

**Additional file 1: Table S1.** Search terms organized by group.

## Data Availability

Not applicable.

## Abbreviations

ABCG2: ATP-binding cassette super-family G member 2
AhR: Aryl hydrocarbon Receptor
ALDH: Aldehyde deshydrogenase
BaP: Benzo(a)pyren
BBP: Benzyl butyl Phthalate
BC: Breast cancer
BMP: Bone morphogenic protein
BP1: Benzophenone-1
BPA: Bisphenol A
cKit: Tyrosine-protein kinase Kit
CSCs: Cancer stem cells
DBP: Di(n-butyl) phthalate
DDE: Dichlorodiphenyldichloroethane
DDT: Dichlorodiphenyltri-chloroethane
DEHP: Di (2-ethylhexyl) phthalate
DMBA: 7,12-Dimethylbenz(a)anthracene
EDC: Endocrine-disrupting chemical
EGFR: Epidermal growth factor receptor
EMT: Epithelial-to-mesenchymal transition
ER: Estrogen receptor
ERK: Extracellular signal-regulated kinase
HCB: Hexachlorobenzene
HDAC: Histone deacetylase
ICAM: Intracellular adhesion molecule
IGF: Insuline growth factor
JAK3: Janus kinase 3
MCF-7: Michigan cancer foundation – 7 cells
MCP1: Chemoattractant protein 1
MMP: Matrix metallopeptidase
nAChR: Nicotinic acetylcholine receptor
NP: Nonylphenol
OCP: Organochlorhine pesticide
Oct4: Octamer-binding transcription factor 4
PAH: Polycyclic aromatic hydrocarbon
PAH: Polycyclic aromatic hydrocarbons
PCB: Polychlorinated biphenyl
PFAA: Perfluoroalkyl acid
PFOA: Perfluorooctanoic acid
PFOS: Perfluorooctane sulfate
PGE2: Prostaglandin E2
PhIP: 2-amino-1-methyl-6phenyl
PI3K: Phosphatidylinositol-3-kinase
POP: Persistent organic pollutant
PTEN: Phosphatase and tensin homolog expression
Ros: Reactive oxygen species
S1PR3: Sphingosine-1-phosphate receptor 3
Sox2: Sex determining region Y-box 2
SP1: Sphingosine 1-phosphate
SPHK1: Sphingosine kinase 1
TCDD: 2,3,7,8-tetrachlorodibenzo-p-dioxin
TE: Trace element
TNBC: Triple negative breast cancer
VCAM: Vascular endothelial cell adhesion molecule
VEGF: Vascular endothelial growth factor
