# Supplementary material for: Environmental chemicals, breast cancer progression and drug resistance
Source: Environ Health. 2020 Nov 17;19:117. doi: 10.1186/s12940-020-00670-2 (PMC7672852; doi:10.1186/s12940-020-00670-2)
Supplement: Supplementary file 1 — Additional file 1: Table S1. Search terms organized by group. [file 12940_2020_670_MOESM1_ESM.docx]

**Table S1: Search terms organized by group**

| **Environmental Pollutants** | | |
| --- | --- | --- |
| Air Pollution  Environmental Pollution  Polycyclic aromatic hydrocarbon  Combustion Products | Gasoline  Diesel  Organic Solvent  Vehicle Exhaust | Dioxin  PAH  Drinking Water  Pesticide |
| **Chemicals identified as mammary carcinogen** | | |
| 1,1,-Dichloroethane  1,2-Dibromo-3-chloropropane (dbcp)1  1,2-Dibromoethane (ethylene dibromide) 1  1,2-Dichloroethane  1,2-Dichloropropane (propylene dichloride) 1  1,2,3-Trichloropropane (1,2,3-TCP) 1  1,3-Butadiene  2-Chloroacetophenone  2,2-bis(Bromomethyl)-1,3-propanediol  2,3-Dibromo-1-propanol  2,4-Dinitrotoluene  2,4-Toluene diisocyanate  Isoeugenol | 3,3'-Dimethoxybenzidine dihydrochloride  1 3,3'-Dimethylbenzidine dihydrochloride  5-Nitroacenapthene  Benzene  C.I. Acid Red 114  C.I. Basic Red 9 monohydrochloride  Chloroprene  Clonitralid  Dichlorvos  Ethylene oxide  Glycidol  Bromochloroacetic acid  Hexachlorobenzene | Indium phosphide  Isoprène  Malachite green  Methylene chloride (dichloromethane) 1  Methyleugenol  Nitromethane  o-Nitrotoluene  o-Toluidine hydrochloride  Ochratoxin A  Sulfallate  Urethane  Acrylamid |
| **Chemicals identified as mammary gland developmental disruptors & other Consumer Product Chemical terms** | | |
| Bisphenol A  BPA  2,2-bis(bromomethyl)-1,3-propanediol  Nonylphenol | DE-71  Phthalate  Consumer products  Polybrominated biphenyl | Personal care producs  Flame retardant  Tris(2,3-dibromopropyl) phosphate |
| **Organochlorine pesticides** | | |
| Aldrin  Dieldrin  Organochlorine | DDE  DDT | Methoxychlor  Heptachlor |
| **Other pesticides** | | |
| Atrazine  Chlordane | Simazine  Captafol | Fenvalerate |
| **Products of combustion** | | |
| 1,3-Dinitropyrene  2-Nitrofluorene  7,12-Dimethylbenz[a]anthracene  1,8-Dinitropyrene  Benzo[a]pyrene  2-amino-1-methyl-6-phenylimidazol[4,5-b]pyridine | 3-Methylcholanthrene  Dibenz[a,h]anthracene  2-Aminoanthracene  1-Nitropyrene  4-Nitropyrene | 6-Nitrochrysene  Dibenzo[def,p]chrysene  PFOA  PFOS |
| **Industrial chemicals** | | |
| 1,4-Dioxane  Hydrazine  Propylene oxide (1,2-Propylene oxide) 1  2,4-Diaminotolunene (2,4-toluene diamine) 1 Nitrobenzene  Styrene  2-Methylaziridine | N-Nitroso-di-n-butylamine  Urethane  4,4'-Methylene-bis(2-choloroaniline)  o-Nitrotoluene  Acrylamide  Ortho-toluidine hydrochloride  Hexachlorobenzène | Vinyl fluoride  Vinyl chloride  Acrylonitrile  Perfluorooctanoic acid  AF-2 (2-(2-furyl)-3-(5-nitro-2-furyl) acrylamide) Propane sultone  Styrene |
| **Metals** | | |
| Toxic metals  Heavy metals  Iron  Cadmium | Tungsten  Copper  Zinc  Chromium | Nickel  Lead |
| **Xenogen Burden** | | |
| **Polychlorinated Biphenyl (PCB)** | | |
| **Alcohol** | | |
| **Cigarette smoke/Tabacco smoke & Nicotine** | | |
